# Betaine in Cereal Grains and Grain-Based Products

**DOI:** 10.3390/foods7040049

**Published:** 2018-03-29

**Authors:** Bojana Filipčev, Jovana Kojić, Jelena Krulj, Marija Bodroža-Solarov, Nebojša Ilić

**Affiliations:** Institute of Food Technology, University of Novi Sad, 21000 Novi Sad, Serbia; jovana.kojic@fins.uns.ac.rs (J.Ko.); jelena.krulj@fins.uns.ac.rs (J.Kr.); marija.bodroza@fins.uns.ac.rs (M.B.-S.); nebojsa.ilic@fins.uns.ac.rs (N.I.)

**Keywords:** betaine, cereals, pseudocereals, gluten-free, stability, cooking, baking, extrusion

## Abstract

Betaine is a non-essential nutrient which performs several important physiological functions in organisms. Abundant data exist to suggest that betaine has a potential for prevention of chronic diseases and that its dietary intake may contribute to overall health enhancement. Several studies have pointed out that the betaine status of the general population is inadequate and have suggested nutritional strategies to improve dietary intake of betaine. Cereal-based food has been implicated as the major source of betaine in the Western diet. This review summarizes the results on the betaine content in various cereals and related products. Attention has been given to the betaine content in gluten-free grains and products. It also discusses the stability of betaine during processing (cooking, baking, extrusion) and possibilities to increase betaine content by fortification.

## 1. Introduction

Betaine (*N*,*N*,*N*-trimethylglycine, glycine betaine) is an organic nitrogenous compound, found for the first time in sugar beet juice (*Beta vulgaris*). 

**Figure 1:**
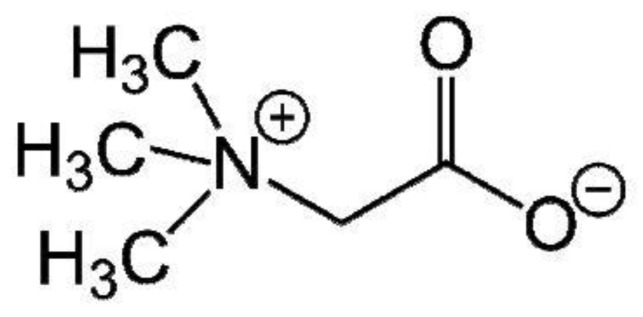
Betaine chemical structure.

Betaine is a zwitterion of quaternary ammonium which is still named trimethylglycine and glycine betaine (Figure 1). It is a methyl derivative of the amino acid glycine ((CH_3_)_3_N^+^CH_2_COO^−^ and molecular weight 117.2). It is characterized as methylamine due to its three free methyl groups [1].

Various analogues of glycine betaine exist in plants: proline betaine (stachydrine), trigonelline, arsenobetaine, betonicine, butirobetaine, ergothionine, propionobetaine, and sulfur analogues. The sulfur analogues are several in type: β-alaninebetaine, dimethylsulfonioacetate, and dimethylsulfoniopropionate (DMSP). The food survey study by de Zwart et al. [2] showed that only some betaine analogues were present in food at appreciable levels (>10 µg/g)—glycine betaine, proline betaine, trigonelline, and DMSP. Slow et al. [3] indicated glycine betaine as dominant in grain products, proline betaine in citruses, and trigonelline in coffee. Most recently, some rare forms of betaine were identified in the grains of most common cereals: pipercolic acid betaine in rye flour and valine betaine and glutamine betaine in flours of barley, rye, oat, durum, and winter wheat [4]. The content of betaine analogues was found to be vastly variable in grains; higher betaine levels seem to be induced by plant growth under stress conditions (drought, salt stress, cold, freezing, hypoxia, etc.) [2,3]. Since the potential health effects of betaine analogues, particularly trigonelline and proline, have not yet been fully resolved, currently only glycine betaine has dietary relevance.

Betaine represents a bioactive compound that has significant physiological functions in the human organism as an osmolite and donor of methyl groups for many biochemical processes. As such, it is indispensable to preserve the health of kidneys, liver, and heart [5]. This compound has an important role in preventing and treating many chronic diseases, among which lowering of plasma homocysteine levels has gained the most attention [5,6,7]. High serum homocysteine levels have been associated with increased risk for cardiovascular diseases (stroke, heart attack, atherosclerosis), cancer, peripheral neuropathy, etc. Moreover, betaine has been shown to improve athletic performance by enhancing muscle endurance [7,8].

Although betaine is not an essential nutrient, it cannot be synthesized in adequate quantities by the human body. Humans may obtain betaine from foods rich in betaine or choline or by oral supplements contained with pure preparations. The main sources of betaine in human nutrition are wheat bran, wheat germ, and spinach [9,10]. Several studies denoted cereal foods as major contributors of betaine [3,11,12,13]. The betaine intake from foods was estimated in a few food surveys (overviewed by Ross et al. [11]) which differ in methodologies used to collect food consumption data, and used different food databases to calculate the betaine intake. Wide gender, national, and international variations were observed [11]. The overall mean betaine intake estimated from these surveys was 131 mg/day [11]. Daily supply of betaine should reach 1500 mg [14] for the manifestation of health effects, so, obviously, the dietary betaine intakes of general populations can be considered to be on the low side. Elderly population (aged over 50 years) or other vulnerable groups (diabetic and homocystinuria patients) may be at a higher risk of inadequate intake. Therefore, many nutritionists advocate for betaine supplementation. Research by Olthof et al. [6] inferred that betaine delivery via food and oral administration of betaine supplement has a similar health effect, where a diet rich in betaine (betaine intake of about 2000 mg/day) had a higher effect on lowering homocysteine than a betaine poor diet (500 mg/day of betaine). It was reported that a betaine-rich meal providing ≈800 mg/day betaine exerted similar acute health effects (increased circulating betaine concentrations and lessened post-methionine load rise in homocysteine) as did the ≈1 g/day supplement [15]. This supports the idea of dietary adjustments to improve the betaine status of general population. These adjustments may span from an effort to include betaine-rich ingredients in the daily individual diet or to imply strategies of food fortification with betaine.

In the US, betaine is recognized as the Generally Recognized as Safe (GRAS) ingredient while in Europe it has been approved by the European Commission for use in food. In 2012, the European Union Regulatory Authority (Commission Regulation (EU) No. 432/2012) [14] permitted the declaration of a health claim on foods containing at least 500 mg betaine per serving, indicating that health effects may be expected if 1500 mg of betaine is administered daily. The medical statement reads: “Betaine contributes to the normal metabolism of homocysteine”. However, this claim should be accompanied with a restriction due to risks associated with excessive intake of betaine: “In order to bear the claim information shall be given to the consumer that a daily intake in excess of 4 g may significantly increase blood cholesterol levels” [14].This work aims to summarize the current findings on the levels of betaine found in cereals and pseudocereals as well as in related products.

## 2. Experimental Methods used in the Analysis of Betaine

In order to determine the content of betaine in food, different methods have been developed. The most common is the liquid chromatography method, but there is no universal method that can be applied to all food matrices. Saarinen et al. [16] analyzed the content of betaine in the chicken liver using a cation exchange column Ca^2+^ and a refractometric detector, although quantification was limited due to poor sensitivity of the detector. Considering physical-chemical properties of betaine, it cannot be analyzed by conventional reversed phase liquid chromatography. Also, betaine has less absorption in the UV–visible spectrum and cannot be detected by a UV detector without derivatization and therefore it is necessary to use reagents for derivatization. De Zwart et al. [2] derivatized a wide range of foods and analyzed betaine with liquid chromatography and UV detector using various columns. Slow et al. [3] have extracted betaine from various foods by using water and dichloromethane, and by derivatization of betaine with 2-naphthacyl trifluoromethanesulphonate. Hefni et al. [17] developed a simple HPLC-UV method in several different food matrices such as spinach, whole grain wheat flour, wheat, and sugar beet, with the help of derivatization on the cation exchange column. More recently, the same group has used the same method to analyze 14 cereal samples, representing different genera and cultivars [18]. Bruce et al. [19] and Ross et al. [11] performed the betaine analysis using LC-MS/MS (liquid chromatography with mass spectrometry) and HILIC (hydrophilic interactions liquid chromatography) column. Bruce et al. [19] developed the LC-MS/MS method for the analysis of 47 blood samples, 32 grains of cereals and cereal fractions, and 51 cereal products. Additionally, Ross et al. [11] analyzed betaine with LC-MS/MS in a wide range of commercially available cereals and cereal fractions. Also, recently Servillo et al. [4] have used LC-ESI-MS/MS for determination of different betaines present in commercial flours of cereals and pseudocereals. Instead of a conventional UV detector for the quantitative determination of betaine in order to avoid derivatization, the evaporative light scattering detector (ELSD) detector is used more recently and as a universal detector that provides a stable base line even in a gradient mode that can detect the majority non-volatile analytes. Shin et al. [20] have proposed a HILIC column in combination with an ELSD detector for betaine analysis. HILIC is an alternative to reverse phase chromatography, namely a type of normal phase chromatography, in which the stationary phase is polar but larger amounts of organic solvents can be used as a mobile phase as opposed to ordinary normal phase chromatography. Kojić et al. have used the HPLC-ELSD system using the HILIC column with isocratic mode of operation [21].

## 3. Cereal Grains as a Source of Betaine

Data on the distribution of betaine in various cereals and pseudocereals are scarce and there is definitely a lack of detailed study. Most data come from various studies that were focused on estimation of betaine dietary intake. Nevertheless, available studies report on wide variations in betaine content in cereals. Different types of cereals may have different amounts of betaine [22]. The following ranges were found by de Zwart et al. [2]: 270–1110 µg/g (dry solids) in wheat flour, and 200–1000 µg/g in oats. More detailed overview of betaine levels in various cereals and pseudocereals from different studies is displayed in Table 1. The displayed data showed that betaine content spanned in wide ranges within the studied grains. According to Corol et al. [22], betaine content in cereals varies depending on multiple factors including genotype and environmental differences such as geographical and/or year-to-year variations and their interactions with genotype. This study revealed a three-fold difference in glycine betaine content within bread wheat genotypes and a 3.8-fold difference across six environments. The highest glycine betaine levels were found in Hungarian wheat grains whereas the lowest in those grown in the UK [22]. Slow et al. [3] and de Zwart et al. [2] indicated that the level of betaine depends on the level of stress under which the crop grows. This is due to osmoprotectant and cryoprotectant function of betaine. For example, growth under drought can cause higher levels of betaine compared to well-watered crops. 

Among glutinous cereals, the highest content of betaine was found in the bran fraction of wheat grain (2300–7200 µg/g) and in the germ (3414 µg/g) (Table 1). In spelt wheat, higher upper betaine levels were detected in comparison to common wheat. Wholegrain spelt flour was much higher in betaine than the wholegrain flour of common wheat (Table 1). Wholegrain flours were mainly higher in betaine when compared to refined flours. Ross et al. [11] estimated that wholegrain flours and products were two to four times higher in betaine in comparison to the refined counterparts. Similar betaine content was found in flour from durum wheat and conventional wheat. In contrast, Ross et al. [11] reported higher levels of betaine in durum semolina in comparison to common, non-refined wheat. 

The most abundant source of betaine was amaranth, a pseudocereal. Raw amaranth grains contained 7420 µg/g betaine which was the highest value determined in a single sample [23]. According to Ross et al. [11] and USDA database [24], quinoa can also be listed as an outstanding source of betaine, having been reported to contain 3930 µg/g and 6300 µg/g betaine, respectively.

## 4. Betaine Content in Cereal-Based Products

The betaine content in cereal products depends on the processing method. Two to four times lower betaine content were found in refined grain products compared to equivalent whole grain products [11]. Betaine content is notably dependent on the loss of bran fraction during processing. The higher the abrasion of aleurone layer, the lower the betaine content in the product. Outstanding betaine levels were determined in wheat bran, up to 7200 µg/g (Table 1). Likes et al. [25] analyzed the betaine contents in different milling streams and reported the lowest betaine level in the cleanest milling fractions. In the study of de Zwart et al. [2], a wide range of different foods was analyzed for betaine content and flour was denoted as an item high in betaine (730 µg/g), however it was not specified the type of flour, except that it was available from retail markets. Betaine ranges in bread, pasta, breakfast cereals and snacks are given in Table 2. As it can be seen, the variation within each product category is high due to versatility of ingredients in product formulations. In each product category, the highest betaine content was reported for wholegrain products or products containing bran or germ. Among breads, rye, spelt, and wholemeal breads were abundant in betaine. Moderate to high betaine contents were reported for pasta products, but it must be noted that mainly uncooked samples were analyzed (Table 2). Breakfast cereals are a mixture of cereal and non-cereal ingredients and the betaine content will depend on the contribution of each ingredient. In the study of Filipčev et al. [23], two samples of commercially available breakfast cereals were analyzed, one of which contained no detectable levels of betaine whereas the other had 471 µg/g (on dry solids). A similar concluded was made by Ross et al. [11] for muesli and muesli bars which were found to contain only low-to-moderate betaine levels. These products were mainly based on oats and contained other low-betaine ingredients such as dried fruits. In contrast to Ross et al. [11], the USDA data [24] report on much wider span of betaine in breakfast cereals, from 7 µg/greaching to as much as 3600 µg/g (on wet weight) betaine.

## 5. Betaine Content in Gluten-Free Cereal Products

Gluten-free products have been generally recognized to be low in betaine content [11,19]. In the majority of commercially available gluten-free products, a very low level of betaine (<50 μg/g) was observed [11]. Table 3 lists the betaine levels reported for commercial gluten-free products from several studies. In the bread and biscuits category, betaine levels ranged from non-detectable to 107 µg/g. Similar findings were reported by Kojić et al. [21], who also found that gluten-free samples (starch, corn extrudates, pasta, cornflakes, and rice) contained no detectable levels of betaine. Gluten-free cereals contained much lower amounts of betaine in comparison to glutenous cereals: corn had 107–304 µg/g betaine [23]; teff and millet between 50–150 µg/g [11], proso millet 280 µg/g [23]. Buckwheat is a frequent ingredient in gluten-free products. According to Ross et al. [11], buckwheat was among those ingredients low in betaine (<20 µg/g) although as high as 390 µg/g betaine was found in buckwheat uncooked pasta (Table 3).

As mentioned earlier, gluten-free ingredients with appreciable amounts of betaine are amaranth and quinoa. Amaranth grain was reported to contain 646–680 µg/g betaine and a remarkable figure of 7420 µg/g betaine in a single sample of raw grains (Table 1). Processed amaranth contained 817–1225 µg/g of betaine (flour) and 669 µg/g (expandate) (Table 1). 

In order to increase betaine levels in gluten-free products and consequently improve dietary intake of betaine among people adhereing to gluten-free and vegan diets, the incorporation of amaranth, quinoa, proso millet, and buckwheat as base ingredients into gluten-free products as well as their enrichment with sugar beet molasses was proposed [11]. Sugar beet molasses can remarkably increase betaine content in some baked products, even when used at fortification levels that do not compromise the sensory properties [26]. It was reported a 43× increase in betaine content of molasses-enriched plain biscuits in comparison to the control biscuit (without molasses) [23]. In gluten-free cookies enriched with molasses at 30% (flour basis), the betaine level was raised ≈64 times [26].

When considering fortification of products with betaine, the challenge is to achieve sufficient delivery of betaine for the claim for lowering blood homocysteine (500 mg/portion). A trial to incorporate betaine in the range from 0.5% to 3% (flour basis) into the formulation of gluten-free biscuits revealed that they were capable of providing from 280 to 1370 mg of betaine per 100 g [27]. The highest fortification level (3%) significantly increased the biscuit spread and contributed to perceiving a weak aftertaste described as burning-like sensations of the tongue and palate which might be due to weak acid reaction of betaine [27]. Rising betaine doses improved color vividness in the biscuits [27]. Similar results were observed in the case of fortifying plain wheat cookies with betaine [28].

## 6. Stability of Betaine in Grain-Based Products

Betaine is known to be a thermostable compound which survives the severe treatment during sugar beet processing (extracting with water, treatment with CaOH_2_ and CO_2_, concentration, crystallization) and almost quantitatively accumulates in molasses [29]. Pure anhydrous betaine decomposes at > 245 °C. Since food processing practices do not employ such high temperatures, betaine losses caused by food thermal treatments were initially not expected [30]. However, some data suggest that certain cooking and baking losses of betaine may exist in spite of its thermostability in the pure form. Being a water soluble compound with a small molecule, it is not unlikely that some betaine losses will occur, depending on the type of food processing and cooking.

Only few studies exist that deal with the stability of betaine in food during processing. De Zwart et al. [2] compared the average betaine content in various food, before and after cooking. They concluded that the level of betaine varied widely, depending on the food and cooking method. The lowest losses (10–14%) were observed with microwave cooking of vegetables (frozen peas, silverbeet) and the highest losses with boiling (43–73%) [2]. In the case of cereal-based food, high betaine losses (76–84%) were detected during pasta boiling which could be attributed to dissolution of betaine in cooking water and its removal upon water draining [2]. Betaine reduction of ≈85% between uncooked and cooked noodles was reported in the USDA database [24] (Table 2). Similar results were confirmed by Ross et al. [11] in cooked pasta and noodles. During baking of scones, de Zwart et al. [2] determined a 17% betaine loss. Similar betaine losses were found after baking gluten-free biscuits fortified with betaine at a 0.5–3.0% level [27]. Somewhat higher baking losses in betaine were reported by Filipčev et al. [28] ranging from 17% to 28.6% in wheat biscuits fortified with betaine at 0.5–3.0% level. Very high betaine losses (>90%) were observed after baking betaine-enriched bread [31]. It was assumed that this loss could be partly due to betaine consumption by baker’s yeast throughout dough fermentation since yeast can use betaine as a source of nitrogen.

During the preparation of extruded snack products enriched with betaine, the influence of extrusion cooking parameters like screw speed, feed flow rate, and feed moisture on the betaine content was analyzed [32]. The most significant influences on betaine content were feed rate and feed moisture content. Under the most extreme conditions applied during the extrusion process betaine losses were from 50–60% [32].

In some cases, an increase in betaine could be observed after thermal treatment as reported for fried and baked falafel [2] and for oatmeal cooked in a microwave oven [11]. The increases were 9, 14, and 31%, respectively. Ross et al. [11] suggested that a plausible explanation of this phenomenon could be the liberation of betaine from food matrix or betaine synthesis throughout heating. So far, it has not been reported that betaine is capable of forming any bonds with matrix components.

## 7. Conclusions

Comparison of betaine levels in cereals from different studies showed that cereals are good sources of betaine. Wheat bran and germs were the most abundant wheat fractions. There were large differences in the betaine contents of different cereals. The wholegrain flours from spelt, rye, and barley showed higher betaine levels in comparison to that of common wheat. Non-glutinous cereals are generally moderately to very low in betaine. The best gluten-free sources of betaine are amaranth and quinoa.

Cereal grain processing may lead to lowering of betaine content, especially if removal of aleurone layers is included. Thermal treatment of cereal products also provokes certain loss of betaine, in spite of its thermal stability on food processing temperatures. Losses are very high if processing involves water removal after cooking or boiling, since betaine is soluble in water. Very high losses were observed during baking of betaine-enriched bread, implying that fermentation by baker’s yeast may be one of the causes but future research is needed to understand the possible mechanisms. 

Fortification of grain-based food with sugar beet molasses even at low or moderate levels considerably raises their betaine content and may be considered as a possible way to increase the functionality of the products.

## Figures and Tables

**Table 1:** Betaine content in different samples of cereals and pseudocereals.

Cereals and Pseudocereals	Betaine	References
	(µg/g Dry Weight)	
Wheat (*Triticum aestivum*)		
raw grain	1150–1320	[18]
	490–574	[23]
bran	5047–5383	[23]
	2717	[21]
	2300–7200	[3]
aleurone	4538–6242	[11]
germ	3414	[11]
wholegrain flour	792	[11]
	730 *	[24]
	604	[19]
	540	[23]
refined flour	718 *	[25]
	700 *	[24]
	415–593	[21,23]
	398	[11]
	180 *	[4]
	141.2	[19]
flour (not specified by origin)	270–1110	[2]
Wheat Emmer (*T. dicoccum*)		
raw grain	830–940	[18]
refined flour	195 *	[4]
Wheat Einkorn (*T. monococcum*)		
refined flour	367.3 *	[4]
Durum wheat (*T. durum*)		
semolina	1227	[23]
	483	[21]
	683	[11]
refined flour	253–303	[23]
	310	[21]
wholegrain flour	713	[11]
	245 *	[4]
Spelt wheat (*T. aestivum* ssp. spelta)		
raw grain	973–2723	[23]
	565–714	[21]
wholegrain flour	1296–1442	[23]
	1370–1430	[18]
refined flour	978	[11]
	522–593	[23]
	410	[21]
Kamut wheat, Khorasan (*T. turgidum* ssp. turanicum)		
raw grains	1100	[24]
Triticale (xTriticosecale)		
raw grain	986–1030	[23]
Rye		
raw grain	2213	[23]
	1530–1760	[18]
	444	[21]
bran	1651	[19]
refined flour	310 *	[4]
wholegrain flour	1500 *	[24]
	1182	[23]
	986	[21]
Barley		
raw grain	460	[18]
raw grain from naked var.	980	[18]
wholegrain flour	776–1023	[23]
	779	[21]
refined flour	250 *	[4]
flour from naked var	424	[21]
	574	[23]
pearled grain	274	[21]
Oats		
raw grain	280	[18]
	388	[21]
raw grain from naked var.	440	[18]
wholegrain flour	310 *	[24]
flour	404–688	[23]
	53 *	[4]
bran	200 *	[24]
	190	[11]
Maize		
raw grain	107–304	[23]
	175	[21]
wholegrain meal	120 *	[24]
degermed meal	4 *	[24]
semolina	3–22	[11]
refined corn grits	37	[11]
flour, enriched	20 *	[24]
refined flour	2.1 *	[4]
bran	184	[21]
	104	[23]
	46 *	[24]
flakes	103–120	[23]
	7–9	[11]
	n.d.	[21]
starch	n.d.	[21]
popped	19	[11]
	n.d.	[21]
Rice		
grain	1–5	[11]
	n.d.	[21]
refined flour	8.4 *	[4]
expanded	n.d.	[21]
starch	n.d.	[21]
Amaranth (*Amaranthus cruentus*)		
raw grain	7420	[23]
	680 *	[24]
	646	[11]
expanded grain	669	[23]
	607	[21]
flour	895–1225	[23]
	871	[21]
Proso millet		
sample type not specified	95–112	[11]
dehulled grain	281	[23]
refined flour	1320 *	[4]
Buckwheat		
wholegrain flour	108	[23]
	7–20	[11]
refined flour	n.d.	[21]
groats, roasted	10 *	[4]
	26 *	[24]
Sorghum		
refined flour	425 *	[4]
Quinoa		
grains	6300 *	[24]
	3042–4428	[11]
	610.8 *	[4]

n.d. not detected; * result expressed on wet weight.

**Table 2:** Betaine content in various grain-based products.

Product	Betaine Content	References
	(µg/g Dry Weight)	
Bread		
rye bread	855–1377	[11]
wholegrain spelt	913	[11]
wholemeal	670–790	[3]
wholegrain	499–781	[11]
	560–620	[3]
multigrain	247–678	[11]
white (refined)	360–520	[3]
	174–287	[11]
various (white, sourdough)	310–590 *	[24]
	380 *	[24]
	579	[19]
wheat tortilla	311	[11]
Pasta		
wholegrain wheat pasta	710–1286	[11]
	375	[19]
pasta, not specified	480–1350	[2]
refined wheat pasta	628–706	[11]
refined wheat (*T. aestivum*) pasta, uncooked	253	[21]
durum wheat pasta, uncooked	188	[21]
one–egg spelt pasta	243–516	[11]
barley pasta	211	[11]
noodles with egg, enriched, uncooked	1300 *	[24]
noodles with egg, enriched, cooked	190 *	[24]
refined couscous	691	[11]
bulghur	1311	[11]
cooked bulghur	830 *	[24]
Breakfast cereals		
ready-to-eat wheat germ, toasted, plain	4100 *	[24]
ready-to-eat wheat bran, toasted	3200 *	[24]
wholegrain rye flakes	1640	[11]
wholegrain wheat-based cereals	732–915	[11]
wholegrain oat and wheat-based muesli	310	[11]
wholegrain oat-based muesli	117–226	[11]
breakfast cereals, not specified	180–300	[21]
muesli bar	171	[11]
wholegrain porridge oats	128–167	[11]
extruded whole grain oat cereals	73–91	[11]
cereal bar	74–75	[11]
various ready-to-eat cereals	7–3600 *	[24]
Snacks, cookies, crackers, crispbread, cakes, pastry		
wholegrain rye crispbread	1428–1527	[11]
frozen, read-to-eat pancakes	690–720 *	[24]
wholegrain wheat crackers	293–649	[11]
crackers, classic, saltines, cheese	340–580 *	[24]
wholegrain wheat rusks	556–564	[11]
wholegrain wheat muffin	437–501	[11]
various commercial cakes	190–480 *	[24]
wholegrain wheat biscuit	425	[21]
Graham cookies	390 *	[24]
doughnuts	270–380 *	[24]
English muffins	220–360 *	[24]
extruded spelt	308	[21]
refined wheat crackers	258–332	[11]
digestive biscuit	271–309	[11]
apple pie, commercial	160 *	[24]
biscuit	4–144	[11]
Danish pastry, fruit enriched	140 *	[24]
plain Danish pastry	81 *	[24]

* Result expressed on wet weight.

**Table 3:** Betaine content in gluten-free products.

Product	Betaine Content (µg/g Dry Weight)	References
Bread and biscuits		
gluten-free crispbread	9–107	[11]
savory biscuits	n.d.–104	[23]
wholegrain gluten-free bread	12–68	[11]
oatmeal biscuits	3	[11]
gluten-free flour enriched with fibers	1	[11]
sweet biscuits	n.d.	[21]
flour mixture for gluten-free bread	n.d.	[21]
gluten-free cookies with almonds, crackers, salty sticks	n.d.	[21]
expanded maize	n.d.	[21]
Pasta		
buckwheat pasta, uncooked	390	[23]
	382	[11]
	175	[21]
maize-based pasta	2–20	[11]
maize and rice-based pasta, uncooked	n.d.	[21]
rice-based pasta, uncooked	n.d.	[21]
Breakfast cereals and related products		
soy bran	182	[21]
unseasoned popcorn	19	[11]
cornflakes	14	[11]
buckwheat flakes	10	[11]
rice-based breakfast cereals	4–5	[11]
expanded rice	n.d.	[21]

n.d. not detected.
